# Ocean science research is key for a sustainable future

**DOI:** 10.1038/s41467-018-03158-3

**Published:** 2018-02-15

**Authors:** Martin Visbeck

**Affiliations:** 1 GEOMAR Helmholtz Centre for Ocean Research Kiel, Wischhofstr. 1-3, 24118 Kiel, Germany; 2 Kiel University, Christian-Albrechts-Platz 4, 24118 Kiel, Germany

## Abstract

Human activity has already affected all parts of the ocean, with pollution increasing and fish-stocks plummeting. The UN’s recent announcement of a Decade of Ocean Science provides a glimmer of hope, but scientists will need to work closely with decision-makers and society at large to get the ocean back on track.

The ocean covers 71% of the Earth’s surface. It regulates our climate and holds vast and in some cases untouched resources. It provides us with basics such as food, materials, energy, and transportation, and we also enjoy the seascape for religious or recreational practices. Today, more than 40% of the global population lives in areas within 200 km of the ocean and 12 out of 15 mega cities are coastal. Doubling of the world population over the last 50 years, rapid industrial development, and growing human affluence are exerting increasing pressure on the ocean. Climate change, non-sustainable resource extraction, land-based pollution, and habitat degradation are threatening the productivity and health of the ocean (Fig. 1). It is in this context that over the last few years, scientists and societal actors have organized a bottom-up movement, which has ultimately led to the United Nations General Assembly proclaiming a Decade of Ocean Science for Sustainable Development (2021–2030). In the process, governments, industry, and scientists have raised awareness of the rapid degradation and over-use of the ocean. The final document from the Rio+20 summit, The future we want^1^, made extensive reference to the ocean, and the Global Ocean Commission articulated the need for more effective global ocean policies^2^. Moreover, the 2030 Agenda for Sustainable Development includes an explicit ocean goal (SDG14)^3,4^ that led to the first-ever UN Ocean conference^5^ to support its implementation. The ambition of the Decade of Ocean Science is to now use this gathering momentum to mobilize the scientific community, policy-makers, business, and civil society around a program of joint research and technological innovation^6^. I see reasons for optimism in four main areas. First, there is a tremendous opportunity to connect ocean sciences more directly with societal actors by promoting integrated ocean observation and solution-oriented research agendas (Fig. 2). Also, rich and poor nations are increasingly engaging in capacity development and resource sharing. And finally, the UN system and coastal states have a unique chance to seriously collaborate in multi-stakeholder processes to advance maritime spatial planning and effective ocean governance.

**Fig. 1:**
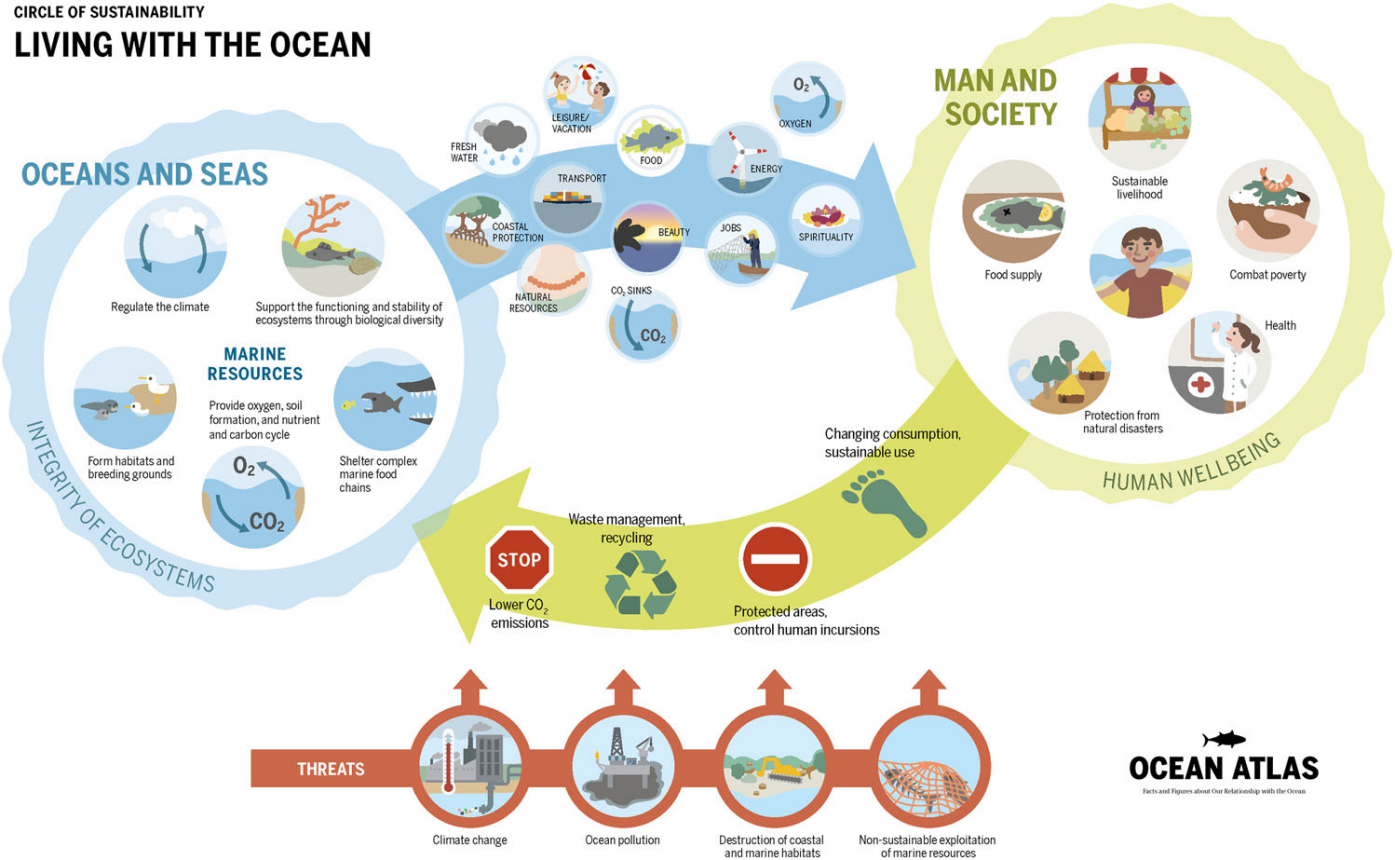
Human–ocean interactions highlighting ocean ecosystem services and their threads (taken from Ocean Atlas, 2017^12^)

**Fig. 2:**
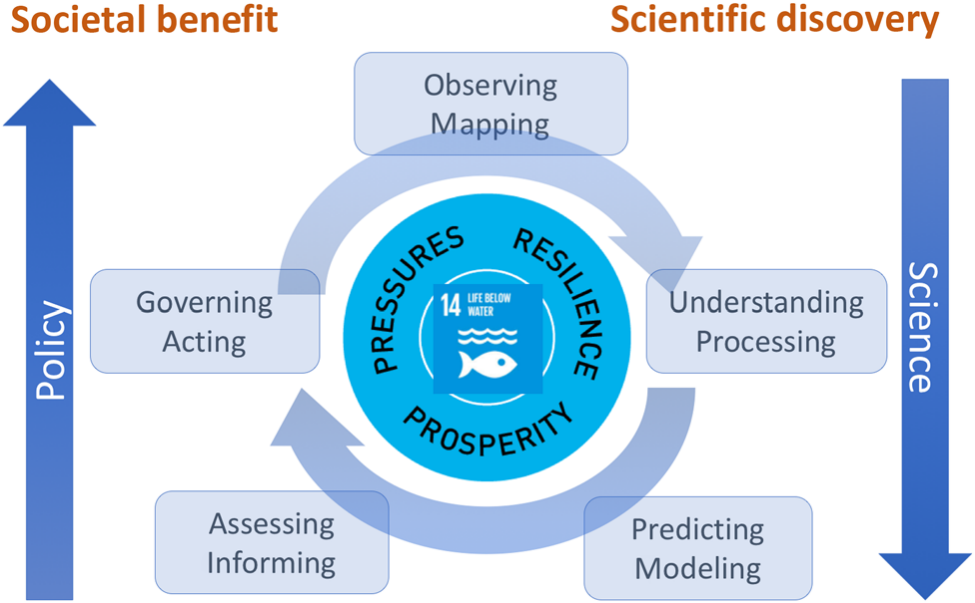
Increased need for ocean information to meet societal needs. Meeting a growing range of societal demands and achieving Sustainable Development Goal 14 (Conserve and sustainably use the oceans, seas, and marine resources) from the 2030 Agenda for Sustainable Development can only be fully realized if all elements of the ocean value chain are resourced adequately and more integrated science agendas are advanced. Figure adapted from ref. 9. Sustainable Development Goal logo ©UNITED NATIONS. All rights reserved

## A more integrated and sustainable ocean observing system

The Decade of Ocean Science will encourage actions towards a more integrated and sustainable ocean observing system to facilitate ocean discovery and environmental monitoring. The vast volume of the ocean and its complex coastlines are neither fully observed nor adequately understood. In particular the deep sea is a frontier of ocean sciences, where internationally coordinated research teams regularly discover new ocean phenomena of profound importance or new organisms and substances for potential future use. Sustained and systematic ocean observations are needed to document ocean change, initialize ocean system models and provide critical information for improved ocean understanding. Advances in ocean robotics and the combination of remote and in situ ocean observations offer new opportunities; and free and open data sharing and multi-stakeholder contributions by governments (rich and poor), the private sector and citizens are opening exciting new dimensions. International efforts, such as the Global Ocean Observing System, the Blue Planet initiative of the Group on Earth Observations and their Framework for Ocean Observing^7^ provide a solid basis and an opportunity for growth. The upcoming decadal conference on ocean observations, OceanObs 19^8^ will provide an excellent opportunity to advance our ocean observing ambitions.

## A solution-oriented integrated ocean science agenda

The Decade of Ocean Science should address both deep disciplinary understanding of ocean processes and solution-oriented research to generate new knowledge. This will support societal actors in reducing ocean pressures, preserving and restoring ocean ecosystems and so safeguard ocean-related prosperity for generations to come. A solution-oriented integrated ocean science agenda can provide innovative ideas, improved assessments and fundamental knowledge in the context of sustainable development and improving human–ocean interactions^9^.

Our rapidly growing, affluent, and more technologically advanced societies are increasingly impacting their local and the global environment, leading to pollution by both chemical and physical wastes. Integrated research is needed to assess the human and environmental risks of ongoing and future types of ocean pollution, to generate new ideas to reduce the ocean pressures by promoting recycling, improved waste management and incentive and governance regimes to encourage more sustainable production and consumption. The most challenging ocean pollutants include: atmospheric carbon dioxide, which causes climate change, ocean warming, ocean acidification, and sea level rise; agricultural fertilizers, which lead to increased primary production but result in ocean deoxygenation; untreated waste water; invasive species; micro and macro plastics, the exponential increase of which has an environmental impact as yet only partially known.

Ocean hazards such as storm surges, harmful algal blooms, or coastline erosion can be devastating for coastal communities. Throughout human evolution civilization has developed strategies to increase our resilience to threats from the ocean. However, the rush for coastal recreation and access to the sea has produced newly built infrastructure that is increasingly vulnerable to ocean extreme events. Hard solutions, such as building walls and levees, could provide some mitigation. However, softer approaches, such as beach nourishment, restoration of mangroves and reef systems, would also provide natural protection and increase resilience to sea-level rise and storm surges. Marine protected areas, natural coastal defences, mining codes, or regulations to limit ocean pollution are all critical elements to safeguard ocean resilience.

Humans have always benefitted from the ocean and its diverse ecosystem services. We often speak of a healthy and productive ocean referring to the desire to maintain the ocean in a prosperous state. The ocean provides food for many, often poor, coastal communities; provides jobs, energy, and raw materials; and enables global trade and recreational and cultural services. The sustainability challenge is achieving long-term ocean prosperity for more affluent societies with a global population approaching 10 billion. Is there sufficient intergenerational will to sustain the overall long-term wealth and well-being of humans by safeguarding ocean resources and ecosystem productivity? What are the trade-offs and synergies between different strategies of marine food production and wild harvesting, different forms of energy harvesting and extraction of materials and ocean restoring zones? New research should develop and flesh out sustainable blue-green growth agendas and link it to efforts in ecosystem protection.

The Decade of Ocean Science should develop a new ocean narrative that can provide context and motivation to reduce ocean pressures, increase ocean resilience, and promote ocean prosperity for generations to come. At the same time, it can provide visibility to existing and new international ocean science programs, such as the new Future Earth Ocean Knowledge–Action network^10^ that aims to connect academic and practical knowledge to address the pressing issues of ocean sustainability using the concept of co-design, co-production, and co-dissemination of ocean sustainability knowledge.

## Global capacity building

The success of the Decade of Ocean Science will critically depend on global capacity building and resource-sharing between countries at different levels of wealth and development. The enormous need for more ocean information at the scientific, governmental, private sector, and public levels demands a step-change in ocean education at all levels. New technology to improve ocean observation, more sustainable ocean resource extraction, and the digital revolution are transforming the ocean sciences and information communities. How can we harness this opportunity? Perhaps new curricula at universities can provide the opportunity to engage a wide range of disciplines in the area of ocean sustainability. Global learning formats such as massive open online courses^11^, open access to ocean information and increased interactions between the academic and societal actor communities are all promising activities. In addition, partnerships between academic and civil society organizations can produce free ocean literacy material, such as the Ocean-Atlas^12^ or the World Ocean Review^13^. However, more engagement at the primary and secondary school levels is urgently needed to promote ocean literacy. Training courses and exchange programs between south–south and north–south ocean actors, as well as courses for ocean professionals, hold tremendous potential to raise ocean awareness and promote better solutions.

## Effective ocean governance

Finally, the Decade of Ocean Science, in conjunction with the 2030 Agenda for Sustainable Development and other international and regional ocean agendas, has shown the need for societal actors to reflect on effective ocean governance. From a regulatory perspective, coastal states can benefit from a systematic, multi-stakeholder assessment and spatial planning procedure. In many parts of the world, each cubic meter of ocean is expected to support several, often conflicting, demands. Spatial planning procedures that take the demands of neighboring countries and the global ocean system into account can help to find more sustainable and equitable regimes of ocean use and access. Science can help in this effort by reflecting on a range of human development scenarios and evaluating how best to sustain ocean prosperity while respecting planetary^14^ and ocean boundaries. A good example of this is the ocean scenario team that is scoping out development pathways to reach SDG14 and ocean-related goals^15^ by 2050 in the context of The World In 2050 project (TWI2050^16^).

The increased awareness of the importance of the ocean to the future of humanity give grounds for cautious optimism and motivation for ambitious multilateral cooperation. The scientific community has been given a stage on which to shine during the Decade of Ocean Science for Sustainable Development. Let us come together, respect our disciplines and agendas but also be ready to embark on an exciting and transformative journey to realize the ocean we need for the future we want.
